# Oral administration of EP4-selective agonist KAG-308 suppresses mouse knee osteoarthritis development through reduction of chondrocyte hypertrophy and TNF secretion

**DOI:** 10.1038/s41598-019-56861-6

**Published:** 2019-12-30

**Authors:** Yasutaka Murahashi, Fumiko Yano, Ryota Chijimatsu, Hideki Nakamoto, Yuji Maenohara, Masahiro Amakawa, Yoshihide Miyake, Hiroyuki Yamanaka, Kousuke Iba, Toshihiko Yamashita, Sakae Tanaka, Taku Saito

**Affiliations:** 1 Sensory & Motor System Medicine, Graduate School of Medicine, The University of Tokyo, 7-3-1 Hongo, Bunkyo-ku, Tokyo, 113-8655 Japan; 2 Bone and Cartilage Regenerative Medicine, Graduate School of Medicine, The University of Tokyo, 7-3-1 Hongo, Bunkyo-ku, Tokyo, 113-8655 Japan; 3 Department of Orthopaedic Surgery, Sapporo Medical University School of Medicine, S-1, W-16, Chuo-ku, Sapporo, 060-8543 Hokkaido, Japan; 4 Research Planning & Collaboration Department, Drug Research Center, Kaken Pharmaceutical Co., Ltd, 14 Shinomiya, Minamigawara-cho, Yamashina-ku, Kyoto, 607-8042 Japan; 5 Pharmacokinetics and Safety Department, Drug Research Center (Shizuoka), Kaken Pharmaceutical Co., Ltd, 301 Gensuke, Fujieda, Shizuoka, 426-8646 Japan

**Keywords:** Molecular medicine, Inflammation

## Abstract

Osteoarthritis (OA) is one of the world’s most common degenerative diseases, but there is no disease-modifying treatment available. Previous studies have shown that prostaglandin E2 (PGE_2_) and PGE2 receptor 4 (EP_4_) are involved in OA pathogenesis; however, their roles are not fully understood. Here, we examined the efficacy of oral administration of KAG-308, an EP_4_-selective agonist, in surgically induced mouse knee OA. Cartilage degeneration and synovitis were significantly inhibited by the KAG-308 treatment. Chondrocyte hypertrophy and expression of tumor necrosis factor alpha (TNF) and matrix metalloproteinase 13 (Mmp13) in the synovium were suppressed in the KAG-308-treated mice. In cultured chondrocytes, hypertrophic differentiation was inhibited by KAG-308 and intranuclear translocation of histone deacetylase 4 (Hdac4) was enhanced. In cultured synoviocytes, lipopolysaccharide (LPS)-induced expression of TNF and Mmp13 was also suppressed by KAG-308. KAG-308 was detected in the synovium and cartilage of orally treated mice. TNF secretion from the synovia of KAG-308-treated mice was significantly lower than control mice. Thus, we conclude that oral administration of KAG-308 suppresses OA development through suppression of chondrocyte hypertrophy and synovitis. KAG-308 may be a potent candidate for OA drug development.

## Introduction

Osteoarthritis (OA), a common degenerative joint disorder in older people, is characterized by progressive cartilage degradation, synovitis and subchondral bone remodeling, which are all associated with OA progression. Various factors are related to its pathogenesis, including mechanical, inflammatory, and metabolic factors that lead to matrix cartilage degradation, aberrant overgrowth of synovial tissues, and subchondral bone marrow lesions^1,2^. Abnormal joint kinematics due to the loss of cartilage, as well as metabolic disturbances, lead to altered subchondral bone elasticity and stiffness. In the guidelines, non-pharmacological modalities such as education and self-management, exercise, and weight loss at first-line treatment are recommended^3,4^. Pharmacological management recommendations included acetaminophen, non-steroidal anti-inflammatory drugs (NSAIDs), and intra-articular injection of corticosteroids. However, there is no disease-modifying treatment that inhibits structural disease progression for OA, and the management of OA is currently limited to pain relief. To overcome the issue, several mouse OA models have been developed using surgical induction of joint instability^5–7^. Experiments of surgical OA models using genetically modified mice have contributed to clarification of various molecules and signaling pathways responsible for its pathophysiology, such as hypertrophic differentiation of chondrocytes characterized by expression of type 10 collagen (Col10a1) and Runt-related transcription factor 2 (Runx2), catabolic enzymes including matrix metalloproteinase 13 (Mmp13), and a disintegrin and metalloproteinase with thrombospondin motifs 5 (Adamts5)^5,6,8–10^. Articular chondrocytes get most of their nutrition from the synovial fluid produced by synoviocytes, and synoviocyte-derived inflammatory factors, including tumor necrosis factor alpha (TNF), which accelerates OA development^11^. Various *in vitro* and *in vivo* studies recently reported novel candidate disease-modifying drugs for OA, including anti-inflammatory agents, antibodies against matrix metalloproteinases, and new pain-relieving drugs^12–18^.

Among these OA-related molecules, several studies have shown that prostaglandin E_2_ (PGE_2_) is increased in the synovial fluid of OA patients and is mechanically regulated in cartilage^19–23^. The function of PGE_2_ in OA is still controversial, because it has both catabolic and anabolic effects in cartilage^19,20,24,25^. In OA cartilage explant cultures, PGE_2_ decreases proteoglycan synthesis^19^. Meanwhile, proteoglycan release from healthy cartilage treated with IL-1β and TNF was further increased by PGE_2_, but proteoglycan synthesis was not^26^. Conversely, PGE_2_ at low concentrations suppresses collagen cleavage via remission of pro-inflammatory genes, and collagenases^24^.

PGE_2_ receptor 4 (EP_4_) is one of four receptor subtypes for PGE_2_. EP_4_ is the most recently discovered subtype of the EP receptors and is insensitive to the other agonists of EP^22^. EP_4_ is coupled with Gsα proteins and activates adenylate cyclase (AC) to increase cAMP, as well as EP_2_^25,27,28^. However, EP_4_ has unique signaling pathways and biological functions including Giα, phosphatidylinositol 3-kinase (PI3K), Epac1, β-arrestin, and β-catenin, which are distinct from those of EP_2_^27,29^. According to recent studies, EP_4_ signaling activates the proliferation and differentiation of mesenchymal stem cells^30–32^. Ho *et al*. showed the enhanced and accelerated repair of damaged muscles following intramuscular administration of PGE_2_ via EP_4_ receptor^30^. *In vitro* studies showed that an EP_4_ agonist suppresses proinflammatory cytokine-induced metalloproteinases^33,34^. The EP_4_ receptor was also up-regulated in human OA cartilage^19,20^. Li *et al*. demonstrated the predominant expression of EP_2_ and EP_4_ receptors in human articular cartilage, and their expression levels are dependent on OA grade^20^. Among various species, amino acid identity of EP4 is relatively maintained, and the sequence homology between human and mouse EP4 is 88%^29^. However, the function of EP_4_ signaling remains unknown, particularly in OA.

EP_4_ is involved in various pathologic conditions, such as autoimmune disease, and the usefulness of an EP_4_ agonist as a treatment for inflammatory bowel disease has been examined in clinical trials^29,35^. Previously, we have reported an orally available EP_4_ selective agonist, KAG-308^36^. In this report, we showed that KAG-308 exerted a suppressive effect on ulcerative colitis (UC) development as well as a mucosal healing effect in a mouse UC model. More recently, it was suggested that the cellular mechanism that suppressed UC by KAG-308 was an anti-inflammatory effect as well as a promoting effect on both proliferation and differentiation of intestinal epithelial cells. Thus, it is expected that an EP_4_ agonist can serve as a novel therapeutic drug for intractable inflammatory diseases such as UC, which need to regenerate intestinal epithelial cells during healing. However, the efficacy of EP_4_ agonists for degenerative joint disorders, such as OA, has not yet been fully evaluated.

Herein, to assess the potential as an oral disease-modifying OA drug (DMOAD) of EP_4_ agonist, we investigated whether KAG-308 could suppress OA development using the mouse surgical model. We orally administered KAG-308 in surgically induced mouse OA for 8 weeks, and then histologically analyzed the development of OA and changes in the expression of OA-related proteins. The local delivery of KAG-308 to the synovium and cartilage was quantified by liquid chromatography–tandem mass spectrometry (LC-MS/MS). We further examined the effects of KAG-308 treatment in the articular chondrocytes, fibroblast-like synoviocytes (FLS), and mesenchymal stem cells (MSC) to reveal the molecular mechanisms of action by KAG-308.

## Results

### Oral administration of KAG-308 suppresses OA development

**Figure 1:**
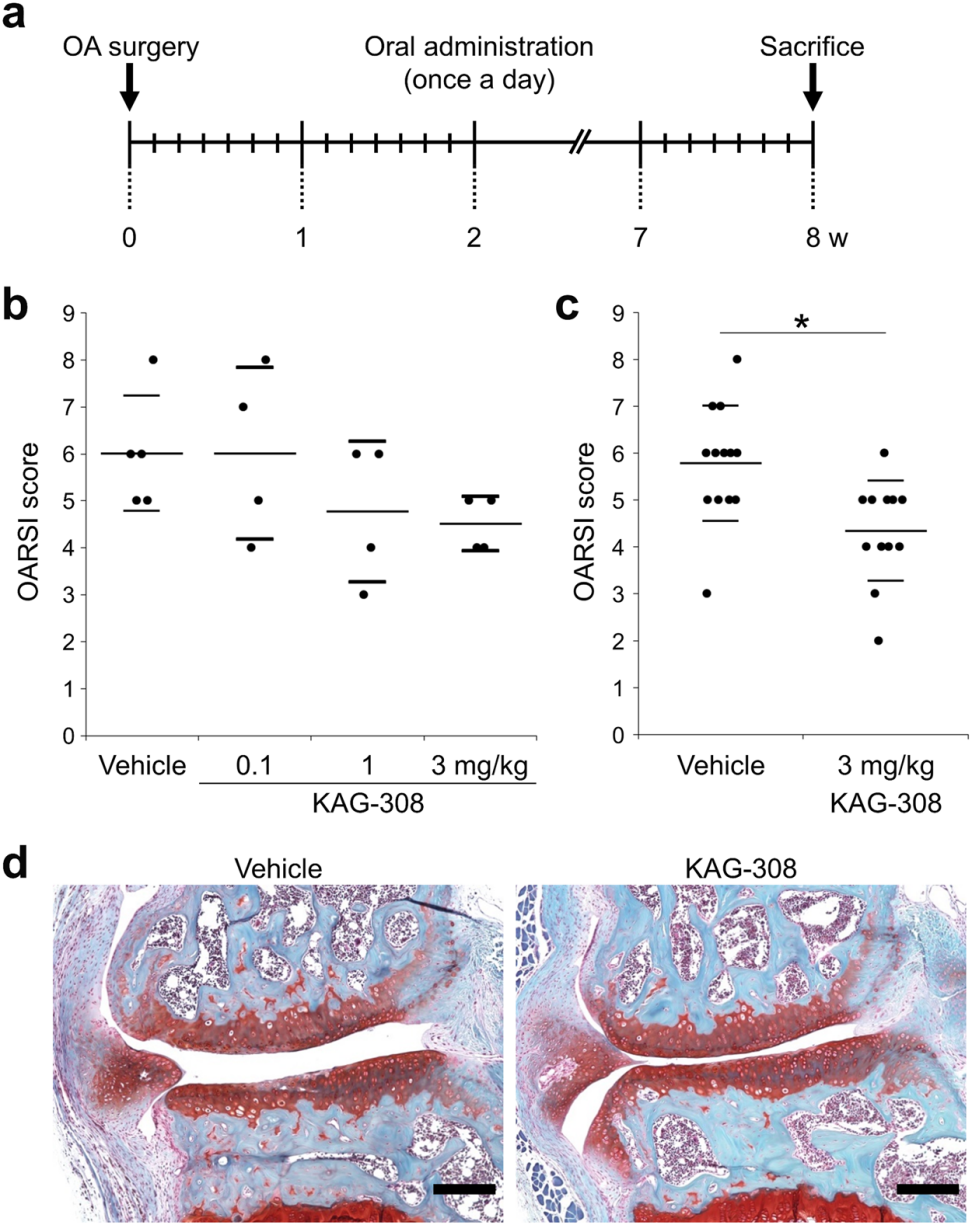
Effects of oral administration of KAG-308 in surgically induced mouse knee osteoarthritis (OA). (**a**) Schematic representation of the surgical procedure for OA induction, oral administrations, and time of sacrifice. Oral treatments were performed once a day from the day after surgery for 8 wks. Quantification of OA development using the Osteoarthritis Research Society International (OARSI) score for (**b**) the first experiment (n = 5 in the vehicle group and n = 4 in the KAG-308 group) and (**c**) the follow-up experiment as a confirmatory phase (n = 13 in the vehicle group and n = 12 in the KAG-308 group). Symbols represent individual mice; long and short bars show the mean and SD, respectively. *P < 0.005 by Welch’s t test. (**d**) Representative safranin-O staining from vehicle and 3 mg/kg KAG-308 groups. Scale bar, 100 µm.

We first examined whether KAG-308 affected the progression of surgically induced OA in mice. Various concentrations of KAG-308 or vehicle were administered orally once a day for 8 weeks from the day after the surgery (Fig. 1a). The Osteoarthritis Research Society International (OARSI) scores indicated that OA development was most suppressed in the 3 mg/kg KAG-308 group (Fig. 1b). We then replicated the treatment with vehicle or 3 mg/kg KAG-308. Histological analyses confirmed that oral administration of 3 mg/kg KAG-308 significantly suppressed OA progression compared with the vehicle control group (Fig. 1c,d), accompanied with marked responsiveness (effect size, 1.24; 95% confidence interval, 0.34–2.05). There was no progression in OA in the contralateral knee after surgery, and no histological difference in cartilage degeneration and volume between the groups treated with vehicle and KAG-308 (Supplementary Fig. S1). Over the 8 weeks, we found no obvious alteration in body weight between the two groups (Supplementary Fig. S2).

### Oral administration of KAG-308 attenuates synovitis

**Figure 2:**
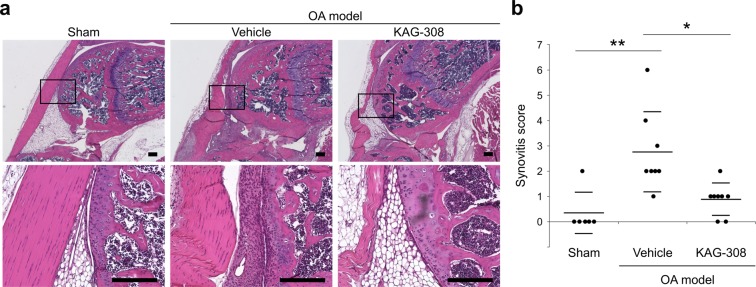
Effect of oral KAG-308 treatment on synovial tissues in surgically induced mouse knee OA. (**a**) Representative hematoxylin and eosin (HE) staining of sagittal knee sections from each experimental group 2 wks after OA induction. Inset boxes in the top panels indicate the regions of the bottom panels. Scale bar, 200 µm. (**b**) Quantification of synovial inflammation in each experimental group (n = 6 in the sham and n = 8 per group in the OA model). Symbols represent individual mice; long and short bars show the mean and SD, respectively. *P < 0.05, **P < 0.005 by ANOVA followed by Tukey’s *post hoc* test.

Oral administration of KAG-308 affected cartilage degeneration; therefore, we evaluated the histological changes in the synovium and the severity of synovitis. The synovitis score was evaluated at 2 weeks after surgery when the synovitis was most remarkable in the present model (Supplementary Fig. S3). In the vehicle group, adipocytes were markedly decreased and fibroblasts filled the synovium (Fig. 2a). Notably, in the KAG-308 group, the lining layer hyperplasia in the synovium and loss of adipocytes were as low as in the sham group (Fig. 2a,b).

### Expression of hypertrophic and catabolic factors is suppressed in cartilage and synovium of KAG-308-treated mice

**Figure 3:**
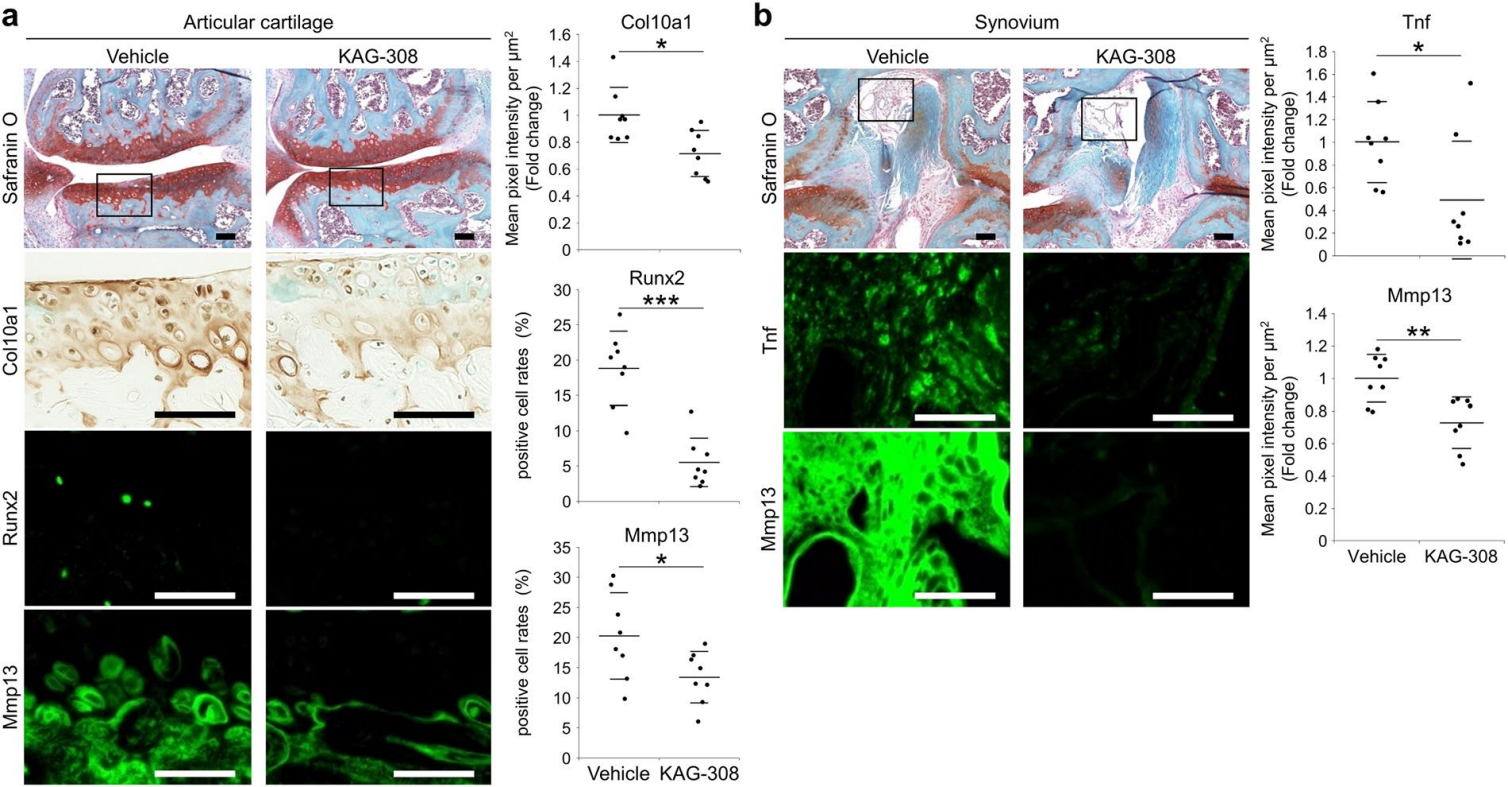
Altered expression of hypertrophic differentiation markers and catabolic factors in cartilage and synovia of KAG-308-treated mice. Safranin-O staining and immunohistochemistry of Runx2, Mmp13, Col10, and Tnf in (**a**) articular cartilage and (**b**) intercondylar synovium of the KAG-308-treated mice 8 wks after OA induction (eight mice per group). Inset boxes in safranin-O staining indicate enlarged images of immunohistochemistry. Scale bar, 50 µm. Right graphs indicate mean pixel intensity per µm^2^ or positive cell rates in the immunohistochemistry. Symbols represent individual mice; long and short bars show the mean and SD, respectively. *P < 0.05, **P < 0.005, ***P < 0.0005 by Welch’s t test, n = 8 mice per group.

We then analyzed the expression of hypertrophic and catabolic factors in articular cartilage and synovium. Immunohistochemistry showed that expression of Col10, Runx2, and Mmp13 was significantly lower in KAG-308-treated cartilage, compared with vehicle-treated cartilage (Fig. 3a). In the synovium, the expression of TNF and Mmp13 was significantly suppressed by KAG-308 treatment (Fig. 3b). These data indicate that KAG-308 inhibited OA development through suppression of chondrocyte hypertrophy, catabolism, or inflammation.

### Local delivery of KAG-308 in cartilage and synovium

To examine the topical delivery of KAG-308, we measured the concentration of KAG-308 in plasma, synovium, and articular cartilage after oral administration of 3 mg/kg KAG-308 by LC-MS/MS. The plasma KAG-308 concentration reached time to peak plasma concentration (T_max_) at 1 hour after oral administration (Table 1). The KAG-308 concentration in the synovium and articular cartilage was less than in plasma; however, the concentration-time profile of KAG-308 was similar to the concentration profile in the plasma. The concentrations in the synovium and articular cartilage were 15.3 ng/g and 6.27 ng/g at the T_max_ (1.0 hour) of plasma KAG-308, respectively. Their tissue molar concentrations were estimated at 33.3 nM and 13.6 nM by assuming a tissue specific gravity of 1, respectively.

**Table 1:** KAG-308 concentration in plasma, synovium, and cartilage after oral administration to mice. The concentrations of KAG-308 were measured at indicated time points after oral administration of 3 mg/kg KAG-308, using LC-MS/MS. The indicated data are three or six mice per time point. N.T., not tested.

		0.5	1	3	6	8	24 hrs
Plasma	ng/ml	24.2 ± 1.4	74.8 ± 89.7	10.8 ± 1.0	18.7 ± 31.0	6.05 ± 0.34	3.03 ± 3.66
	(nM)	(52.6 ± 3.0)	(162 ± 195)	(23.4 ± 2.1)	(40.6 ± 67.4)	(13.1 ± 0.8)	(6.58 ± 7.93)
Synovium	ng/g	N.T.	15.3 ± 20.4	1.37 ± 1.12	3.45 ± 8.45	N.T.	N.T.
	(nM)		(33.3 ± 44.3)	(2.97 ± 2.44)	(7.48 ± 18.33)		
Cartilage	ng/g	N.T.	6.27 ± 3.76	3.87 ± 5.69	6.03 ± 7.27	N.T.	N.T.
	(nM)		(13.6 ± 8.2)	(8.41 ± 12.36)	(13.1 ± 15.8)		

### cAMP signaling pathway is activated by KAG-308

**Figure 4:**
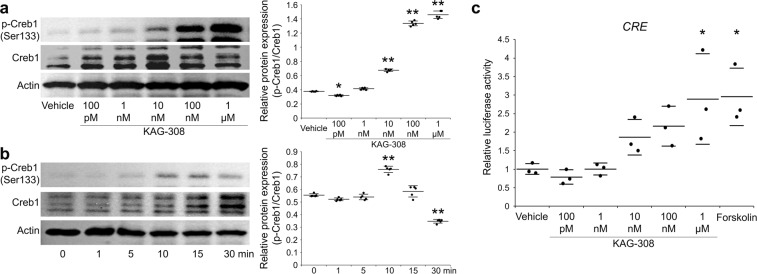
Activation of cAMP signaling pathway by KAG-308. (**a**) Immunoblotting of phosphorylated Creb1 on Ser133, Creb1, and Actin in mouse fibroblast-like synoviocytes (FLS) 15 min after treatment with different concentrations of KAG-308. Right graph indicate quantitative densitometry analysis of the left immunoblot (n = 5). p-Creb1 values were normalized to Creb1. Long and short bars show the mean and SD, respectively. *P < 0.05, *P < 0.005 vs vehicle by ANOVA followed by Dunnett’s *post hoc* test. (**b**) Time course of the phosphorylation of Creb1 on Ser133 in mouse FLS treated with 10 nM KAG-308. Right graph indicates quantitative densitometry analysis of the left immunoblot (n = 5). p-Creb1 values were normalized to Creb1. Long and short bars show the mean and SD, respectively. **P < 0.005 vs 0 min by ANOVA followed by Dunnett’s *post hoc* test. (**c**) Luciferase activities in SW1353 transfected with the *CRE* reporter vector. KAG-308 was added 24 hrs after the transfection, and the cells were cultured for additional 24 hrs. Forskolin (10 µM) was used as positive control. Symbols represent individual points; long and short bars show the mean and SD of three wells per group, respectively. *P < 0.05 vs vehicle by ANOVA followed by Dunnett’s *post hoc* test.

To confirm an EP_4_-agonistic effect of KAG-308, we examined the activation of the cAMP signaling pathway. Ser133 phosphorylation of cAMP response element binding protein 1 (Creb1), a representative transcription factor downstream of the cAMP signaling pathway, was clearly increased by over 1 nM of KAG-308 in a dose-dependent manner (Fig. 4a). Phosphorylated Creb1 proteins were most increased at 10 min after 10 nM KAG-308 treatment (Fig. 4b). Furthermore, we confirmed that KAG-308 similarly enhanced the luciferase activity of a CRE reporter vector, as well as Forskolin, an adenylyl cyclase activator (Fig. 4c).

### Nuclear translocation of histone deacetylase 4 is promoted by KAG-308

**Figure 5:**
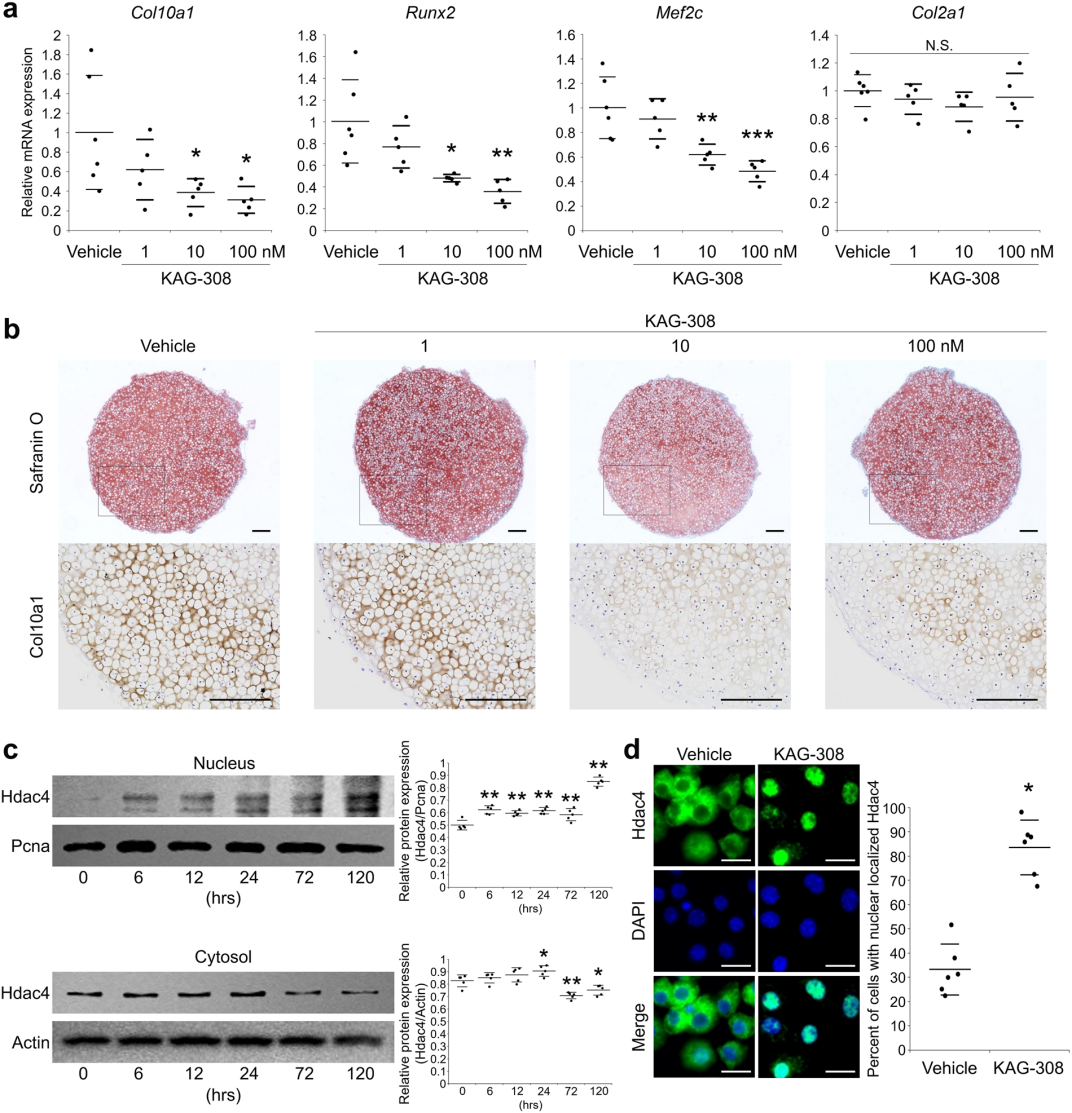
Effects of KAG-308 on chondrocyte hypertrophy *in vitro*. (**a**) mRNA levels of *Col10a1*, *Runx2*, *Mef2c*, and *Col2a1* in pellet culture of mouse articular chondrocytes treated with indicated concentration of KAG-308 for 2 wks. Symbols represent individual pellets; long and short bars show the mean and SD, respectively. *P < 0.05, **P < 0.005, ***P < 0.0005 vs vehicle by ANOVA followed by Dunnett’s *post hoc* test. (**b**) Safranin O staining and Col10a1 immunostaining of cultured pellets of mouse articular chondrocytes treated with KAG-308 for 2 wks. Inset boxes in safranin-O staining indicate enlarged images. Scale bar, 200 µm. (**c**) Immunoblotting of Hdac4 in nuclear and cytosolic fractions from mouse articular chondrocytes in a time course with 10 nM KAG-308 treatment. Right graphs indicate quantitative densitometry analysis of the immunoblots (n = 5). Hdac4 values were normalized to Pcna (nuclear) or Actin (cytosol). Long and short bars show the mean and SD, respectively. *P < 0.05, **P < 0.005 vs 0 hour by ANOVA followed by Dunnett’s *post hoc* test. (**d**) Immunocytochemistry of Hdac4 in mouse articular chondrocytes after 4-d-culture in BMP-2-containing medium with vehicle or 10 nM KAG-308. Right graph shows the percent of cells with nuclear-localized Hdac4. Symbols represent independent slides; long and short bars show the mean and SD, respectively. *P < 0.0005 by Welch’s t test. Scale bar, 20 µm.

We studied the molecular mechanisms underlying the amelioration of OA development by KAG-308. We first examined the effects of KAG-308 against hypertrophic differentiation. In pellet cultures of chondrocytes, KAG-308 dose-dependently decreased the expression of hypertrophic marker genes *Col10a1*, *Runx2*, and myocyte enhancer factor 2C (*Mef2c*), while type 2 collagen (*Col2a1*), a representative cartilage matrix gene, was not changed (Fig. 5a). In histological analysis, protein levels of Col10a1 were suppressed by KAG-308, but proteoglycan content, determined by safranin-O staining, was not changed (Fig. 5b).

Previous studies have shown that the cAMP pathway enhances nuclear translocation of histone deacetylase 4 (Hdac4) and suppresses chondrocyte hypertrophy^37–40^; thus, we hypothesized that KAG-308 may exert anti-hypertrophic effects via the cAMP/Hdac4 pathway. Immunoblot analyses showed that the Hdac4 level in the nuclear fraction was gradually increased after KAG-308-treatment, while Hdac4 in the cytoplasmic fraction decreased by contrast (Fig. 5c). To further confirm translocation of Hdac4 from the cytosol into the nucleus, we performed the immunocytochemistry of chondrocytes treated with or without KAG-308. KAG-308 markedly enhanced the nuclear translocation of Hdac4 96 hours after the treatment (Fig. 5d). Quantification of nuclear translocation indicated that Hdac4 was detected in the nucleus of only 33% of vehicle-treated chondrocytes, while 83% of KAG-308-treated cells were nuclear-Hdac4 positive (Fig. 5d).

### TNF secretion from synoviocytes is suppressed by KAG-308

**Figure 6:**
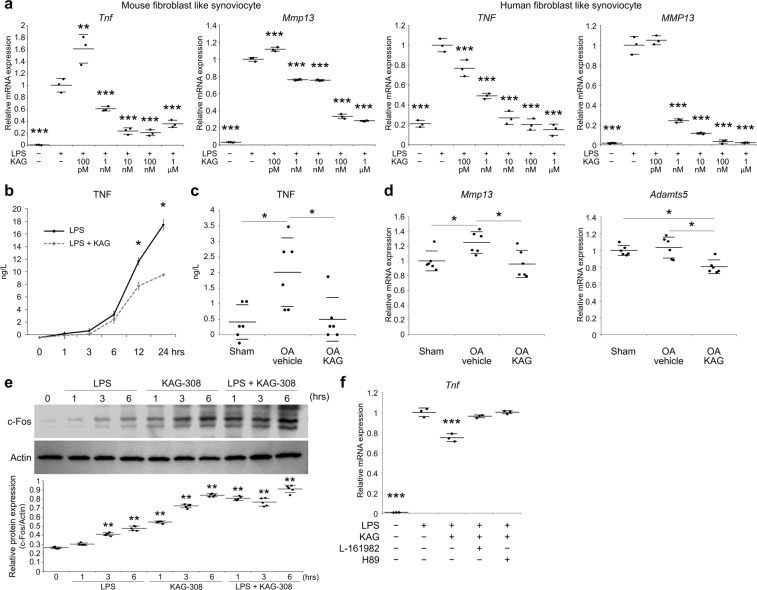
Effects of KAG-308 on TNF secretion from FLS. (**a**) mRNA levels of *Tnf* and *Mmp13* in mouse and human FLS. After 8 hr-starvation, cells were simultaneously treated with 10 ng/ml LPS and KAG-308 for 6 hrs. Symbols represent individual points; long and short bars show the mean and SD of three wells per group, respectively. *P < 0.05, **P < 0.005, ***P < 0.0005 vs LPS + /KAG-308− by ANOVA followed by Dunnett’s *post hoc* test. (**b**) TNF levels in culture media of mouse FLS at 0, 1, 3, 6, 12, and 24 hrs after LPS treatment with or without 10 nM KAG-308 determined by ELISA. Data are expressed as the mean of three wells per group. Error bars indicate SD. *P < 0.05 at each time point by Welch’s t test. TNF levels in the culture media of synovial tissue obtained from sham or OA mice treated with vehicle or 3 mg/kg KAG-308 for 2 wks (**c**), and mRNA levels of *Mmp13* and *Adamts5* in mouse articular chondrocytes after 24-hr-culture in the conditioned medium from synovium (**d**). Symbols represent individual specimens; long and short bars show the mean and SD, respectively. *P < 0.05 by ANOVA followed by Tukey’s *post hoc* test. (**e**) Immunoblotting of c-Fos and Actin at 0, 1, 3, and 6 hrs after treatment with LPS and/or 10 nM KAG-308. Lower graph indicates quantitative densitometry analysis of immunoblots (n = 5). c-Fos values were normalized to Actin. Long and short bars show the mean and SD, respectively. **P < 0.005 vs 0 hour by ANOVA followed by Dunnett’s *post hoc* test. (**f**) mRNA levels of *Tnf* in mouse FLS treated with LPS, KAG-308 (10 nM), and either EP_4_ antagonist L-161982 (10 µM) or PKA inhibitor H-89 (10 µM) for 6 hrs. KAG-308 and LPS were simultaneously added one hr after L-161982 or H-89 treatment. Symbols represent individual points; long and short bars show the mean and SD of three wells per group, respectively. ***P < 0.0005 vs LPS + /KAG-308−/L-161982−/H-89− by ANOVA followed by Dunnett’s *post hoc* test.

We next studied the anti-inflammatory effect of KAG-308 *in vitro* and *ex vivo*. In mouse and human FLS, lipopolysaccharide (LPS)-induced expression of TNF and Mmp13 was suppressed by KAG-308 in dose-dependent manner (Fig. 6a). TNF secretion from mouse FLS, determined by enzyme-linked immunosorbent assay (ELISA), gradually increased up to 24 hours after the LPS induction (Fig. 6b), and KAG-308 treatment significantly suppressed the secretion at 12 and 24 hours (Fig. 6b). To confirm suppression of TNF secretion by KAG-308, we obtained the synovial tissues from knee joints of sham and OA model mice that were orally treated with vehicle or KAG-308. The amount of TNF released from the synovium of vehicle-treated OA mice was significantly increased compared with sham mice; however, in the media from the synovium of KAG-308-treated OA mice, the TNF level was decreased to a level similar to the sham mice (Fig. 6c). We further cultured primary mouse chondrocytes using conditioned medium (CM) obtained from cultures of synovial tissues. Expression of Mmp13 and Adamts5 was downregulated after culture with the CM from the synovium of KAG-308-treated mice (Fig. 6d).

We then searched for molecules involved in the suppression of TNF secretion by KAG-308. We focused on c-Fos, because c-Fos protein accumulates after the activation of the cAMP/PKA signaling pathway, and reduces the recruitment of p65/RelA to the *Tnf* promoter^41^. Immunoblots showed that c-Fos protein was increased by KAG-308 treatment at 3 and 6 hours, and the increase was enhanced by LPS stimulation (Fig. 6e). Furthermore, the suppression of TNF secretion by KAG-308 was abrogated by the EP_4_ antagonist L-161982 or the PKA inhibitor H89 in mouse FLS (Fig. 6f).

### Proliferation and chondrogenic differentiation of MSC are accelerated by KAG-308

We finally examined the effect of KAG-308 on human MSC, because somatic stem cells are involved in OA pathogenesis and cartilage regeneration^42–44^. Cell proliferation assays showed that KAG-308 accelerated the proliferation of MSC (Supplementary Fig. S4a). In pellet cultures of MSC, KAG-308 enhanced the expression of *COL2A1* and *Aggrecan* (*ACAN)* in a dose-dependent manner (Supplementary Fig. S4b). Safranin-O staining and immunostaining of COL2A1 showed enhanced cartilage matrix synthesis following KAG-308 treatment (Supplementary Fig. S4c). We performed the same experiments using MSC from different five donors and showed that cell proliferation was similarly enhanced in the MSC from all donors. MSC from three donors displayed similar chondrogenic responses to the representative results shown in Supplementary Fig. S4b,c, but MSC from other two donors did not.

## Discussion

In the current study, we showed that oral administration of KAG-308, an EP_4_-selective agonist, prevented the structural disease progression associated with synovial inflammation of surgically induced mouse OA. Histological analyses indicated that both chondrocyte hypertrophy and expression of TNF and Mmp13 in synovium were suppressed in the KAG-308-treated mice. Hypertrophic differentiation was inhibited by KAG-308 in cultured chondrocytes, and intranuclear translocation of Hdac4 was enhanced. In cultured synoviocytes, LPS-induced expression of TNF and Mmp13 was also suppressed by KAG-308. Notably, TNF secretion from synovium of KAG-308-treated mice was significantly lower than in control mice. These data suggest that KAG-308 regulates OA development through suppression of chondrocyte hypertrophy and synovitis. Many of the candidate OA drugs being studied are locally administered, and oral administration is useful in view of the clinical application. KAG-308 is found to be transferred to intra-articular tissues including synovium and articular cartilage at an effective concentration by daily oral administration.

In the past decade, several studies have reported that EP_4_ mRNA is abundant not only in human leukocytes and spleens, but also in synovial fibroblasts^29,45^. Formerly, prostaglandins were well-known mediators of inflammation^46^. Meanwhile, it is currently recognized that the EP_4_ signaling exerts anti-inflammatory effects in immune systems^27,29,47^. LPS-induced PGE_2_ acts on macrophages as an inhibitor of TNF production through EP_2_ and EP_4_ in an autocrine manner, suggesting the multifaceted roles of prostaglandins in the regulation of inflammation^48^. In the present study, KAG-308 suppressed the LPS-induced increase of *TNF* and *Mmp13* in FLS (Fig. 6a). Oral administration of KAG-308 inhibited the synovial inflammation and suppressed the upregulation of TNF and Mmp13 protein in synovium. Synovial inflammation is a pivotal element of osteoarthritis, and the cartilage degradation process occurs via pro-inflammatory cytokines and the production of MMPs from synovium^49,50^. The anti-inflammatory effect of KAG-308 in synovia is probably associated with suppression of OA development.

Koga *et al*. demonstrate that the induction of c-Fos by cAMP accumulation suppressed LPS-induced TNF via reduction of p65/RelA recruitment to the Tnf promoter^41^. An EP_4_ receptor couples to the G protein and activates adenylyl cyclase, resulting in the enhanced production of intracellular cAMP^29^. In the present study, KAG-308 increased c-Fos protein and suppressed TNF secretion from FLS, which was negated by the PKA inhibitor H89 as well as the EP_4_ antagonist L-161982 (Fig. 6e,f). Considering that activation of the NF-κB signaling pathway leads to OA development through the induction of catabolic enzymes including Mmp13 and Adamts5^7,51,52^, KAG-308 may suppress these enzymes and cartilage degeneration via c-Fos accumulation in chondrocytes.

Previous studies indicate that Hdac4 is one of the key regulators of chondrocyte hypertrophy. Mef2c is a representative transcription factor, which promotes hypertrophic differentiation^53^. The cAMP-protein phosphatase 2A axis suppresses chondrocyte hypertrophy by promoting the nuclear translocation of Hdac4, which inhibits the expression of Mef2c^37–40^. Yahara *et al*. demonstrate that an intra-articular injection of Sik3 inhibitor prevents chondrocyte hypertrophy and suppresses OA progression via activation of Hdac4^14^. The molecular mechanisms underlying the KAG-308 effects on chondrocyte hypertrophy and synovitis are different, and both are favorable for OA prevention.

The availability and activity of MSC contribute to normal development and homeostasis of joints^42^. MSC or progenitors are clearly capable of mobilizing in response to the stress or injury, and may be involved in the remodeling or repair of cartilage through paracrine activity^43^. MSC isolated from patients with end-stage OA have less proliferative and differentiation activity compared with healthy MSC^44^. In the present study, KAG-308 significantly enhanced the cell proliferation of MSC from all five donors, while it promoted chondrogenic differentiation of MSC from three of five donors (Supplementary Fig. S4). Moreover, we used bone marrow MSC for these experiments, but we did not examine the proliferation and chondrogenesis of synovium-derived MSC in the presence of KAG-308. Taken together, these data are preliminary and we could not confirm the *in vivo* effects on MSC; however, KAG-308 may be involved in the anabolic effects of MSC, as well as favorable effects on chondrocytes and synoviocytes.

The roles of PGE_2_ in articular joints and OA pathophysiology have not been fully understood. Several studies have shown that PGE_2_ is increased in the synovial fluid of OA patients at the concentration of 10–100 pg/ml (about 28–280 pM) using ELISA or LC-MS/MS analysis^20,21^. Tchetina *et al*. reported that PGE_2_ showed an anti-catabolic effect with concentrations of 1–100 pg/mL in explants of human OA knee articular cartilage^24^. Conversely, PGE_2_-induced catabolism in chondrocytes as pro-inflammatory mediators at higher concentration, e.g., 10 µM^15,19,20^. In the current study, the concentration of KAG-308 in intra-articular tissues was higher than 10 nM 1 hour after the oral administration of 3 mg/kg KAG-308 (Table 1). Meanwhile, KAG-308 exerted not only the anti-inflammatory effect on synoviocytes with concentrations higher than 10 nM but also the suppressive effect on chondrocyte hypertrophy with concentrations higher than 1 nM in *in vitro* experiments. Therefore, when KAG-308 is exposed to intra-articular tissues with a concentration higher than 10 nM, it may exert the suppressive effects on OA. Meanwhile, *in vitro* experiments showed that100 nM KAG-308 had better effects against chondrocyte hypertrophy and synovitis than 10 nM, but we did not examine higher doses than 3 mg/kg. Considering that PGE_2_ exerts paradoxical effects in chondrocytes according to concentration, it is necessary to examine a greater range of KAG-308 concentrations in further *in vivo* studies.

In the current study, we initiated the oral administration of KAG-308 at the time of the OA induction. Treatment with KAG-308 suppressed OA development; however, we could not determine whether KAG-308 could induce cartilage regeneration in OA joints. Another limitation is that we harvested primary samples from mice of different ages. Primary chondrocytes are usually harvested from 5-day-old mice, as previously described^54^, because it is difficult to obtain proliferative primary chondrocytes from adult mice. Meanwhile, to collect synovial tissues, it is necessary to perform OA surgery at 10 weeks due to synovial size.

In conclusion, we demonstrated that oral administration of KAG-308 prevented surgically induced mouse knee OA through suppression of chondrocyte hypertrophy and synovitis. The amino acid sequence of EP_4_ is relatively conserved among various species, and the sequence homology between human and mouse EP_4_ is 88%^29^. In the present study, the effects of KAG-308 are replicated in human cells. Considering that KAG-308 can be delivered to chondrocytes and synovium via oral administration, it may be a potent candidate for a DMOAD targeting EP_4_. Since KAG-308 is a compound that has been evaluated in human UC clinical trials, it is advantageous to apply it to human OA.

## Methods

### Surgical induction of osteoarthritis in mice and oral administration of KAG-308

All animal experiments were authorized by the Animal Care and Use Committee of The University of Tokyo. We have complied with all relevant ethical regulations. C57BL/6J male mice were housed in plastic cages with free access to drinking water and a pellet based diet. Experimental OA was induced in 8-wk-old male mice, as described previously^6,8^. Briefly, under general anesthesia, the medial collateral ligament and the medial meniscus of the right knee were resected under a microscope. Mice were administered KAG-308 in distilled water orally once a day from the day after surgery for 8 weeks (Fig. 1a). Control mice were administered 10 mL/kg distilled water (vehicle) following the same regime. To examine the effective doses of KAG-308, we first performed OA surgery for 4 to 5 mice per group, and treated them orally with vehicle or various doses of KAG-308 (0.1 mg/kg or 1 mg/kg or 3 mg/kg) for 8 weeks after surgery (Fig. 1b). OA severity was assessed at 8 weeks after surgery. OA development was most suppressed in the 3 mg/kg KAG-308 groups in the first examination; therefore, we replicated oral administration of vehicle or 3 mg/kg KAG-308 using 8 mice per group for 8 weeks. We additionally performed OA surgery in eight mice for histological analysis of synovitis and six mice for the evaluation of TNF production from the synovium during OA progression in each group, and analyzed them after two weeks of treatment. To compare with knees without the OA induction, a sham operation was performed in six mice for histological analysis of synovitis and in another six mice for the evaluation of TNF production from the synovium using the same approach without ligament transection or meniscectomy.

### Histology and immunohistochemistry

The samples were fixed in 4% paraformaldehyde, decalcified in 10% ethylenediaminetetraacetic acid (EDTA), and embedded in paraffin. Hematoxylin and eosin (HE) and safranin-O/fast green staining were performed according to standard protocols. Synovitis was scored using HE staining of the sagittal knee section at 2 weeks after surgery as previously described^55^. For cartilage evaluation, safranin-O staining of the coronal knee section at 8 weeks after surgery was quantified using the OARSI scoring system for grading of the severity of knee OA^56^. The scores of the femur and tibia were summed and presented as the OARSI score for each sample. For quantitative outcome data, Cohen’s d effect size was applied to estimate the overall effect size of KAG-308 in mice models of OA with a power of 0.80 and an alpha of 0.05 (<0.2: not clinically relevant; >0.2: small; >0.5: moderate; >0.8: large; >1.2: very large). For immunohistochemistry, sections were incubated with the following antibodies diluted in blocking reagent: Col10a1 (1:400, 14–9771–80; eBioscience, San Diego, CA), Runx2 (1:100, sc-10758; Santa Cruz Biotechnology, Dallas, TX), Mmp13 (1:250, MAB13426; Merck Millipore, Darmstadt, Germany), TNF (1:100, ab6671; Abcam, Cambridge, UK), HDAC4 (1:100, sc-46672), and Col2a1 (1:200, MAB8887; Merck Millipore). For immunofluorescence, we used a CSA II Biotin-Free Catalyzed Amplification System (K1497, Agilent Technologies, Santa Clara, CA) and applied VECTASHIELD Mounting Medium with DAPI (Vector Laboratories, Burlingame, CA). Six to eight representative individual samples from each treatment group were used to measure the expression level of target protein in articular cartilage, intercondylar synovium (between the anterior cruciate ligament and posterior cruciate ligament), or cell culture experiments. We analyzed the percentage of positive cells/DAPI or the mean pixel intensity per µm^2^ for semi-quantification. These procedures were performed by using the BZ-X analyzer software (Keyence, Osaka, Japan). The mean pixel intensity per µm^2^ in control mice was defined as “1”.

### Measurement of the KAG-308 concentrations in intra-articular tissues and plasma

Synovial tissue, articular cartilage of knee joints, and blood were collected from the normal mice after single oral administration of 3 mg/kg KAG-308. Sampling points of intra-articular tissues were 1, 3, and 6 hours after administration. Sampling points of blood were 0.5, 1, 3, 6, 8, and 24 hours after administration. One animal was used at each sampling point. The number of animals at each time point was set to consider reproducibility [n = 3 (0.5, 8 and 24 hours) or n = 6 (1, 3, and 6 hours)]. Synovial tissues and cartilage were washed with saline to remove adhered blood, followed by precipitation with methanol. The plasma was obtained by centrifugation of blood. The KAG-308 concentration in intra-articular tissues and plasma were measured by LC-MS/MS. The LC-MS/MS analysis was conducted using Shimazu Nexera HPLC system (Shimadzu Scientific Instruments, Inc.) and ABSciex QTRAP5500 Mass Spectrometer (ABSciex Corp) with Electrospray Ionization (ESI) and negative Multiple Reaction Monitoring (MRM) Scan.

### Isolation and culture of mouse articular chondrocytes and fibroblast-like synoviocytes

Primary articular chondrocytes were isolated from 5-day-old mice according to the standard protocol using collagenase D (Roche Applied Science, Penzberg, Germany)^54^. Isolated chondrocytes were cultured in DMEM supplemented with 10% fetal bovine serum (FBS) and 1% penicillin-streptomycin (PS). To isolate mouse FLS, synovial tissues were harvested from the intra-articular synovium of 10-week-old male mice and digested with 0.1% collagenase D for 1 hour at 37 °C with shaking as previously described^57^. Cell suspensions were centrifuged, resuspended and seeded onto culture dishes in DMEM supplemented with 10% FBS, and 1% PS at 37 °C, 5% CO_2_, 3% O_2_ to avoid oxidative DNA damage which prevents the cell proliferation^58^. In the immunoblotting analysis of phosphorylated Creb1 using mouse FLS, cells were analyzed at 15 min after treatment with different concentrations of KAG-308 to examine the minimum concentration of cAMP activation. We also analyzed the time course of Creb1 phosphorylation using 10 nM KAG-308 which was the synovial concentration in the mouse OA model treated with oral administration of 3 mg/kg KAG-308. In the analysis of LPS-induced over expression of TNF and Mmp13 using mouse FLS, cells were simultaneously treated with 10 ng/ml LPS (Sigma-Aldrich, St. Louis, MO) and various concentrations of KAG-308 for 6 hours to examine the minimum KAG-308 concentration required for anti-inflammatory effects. The concentration of LPS that could induce TNF was 10 ng/ml, as previously reported^41^. In the immunoblotting analysis of c-Fos using mouse FLS, cells were simultaneously treated with 10 ng/ml LPS and 10 nM KAG-308, and were then lysed at 0, 1, 3, and 6 hours after treatment. In the analysis of co-incubation of EP4 antagonist or PKA inhibitor using mouse FLS, 10 nM KAG-308 and 10 ng/ml LPS were simultaneously added 1 hour after EP_4_ antagonist L-161982 (Sigma-Aldrich) or PKA inhibitor H89 (Cayman Chemical, Ann Arbor, MI). The concentration of L-161982 that could inhibit EP4 signaling was 10 µM, as previously reported^59^, and the concentration of H89 that could inhibit PKA activity was 10 µM, as previously reported^60^. In the analysis of LPS-induced TNF secretion from mouse FLS, the media was collected at 0, 1, 3, 6, 12 and 24 hours after treatment with 10 ng/ml LPS and 10 nM KAG-308, and stored at −80 °C until ELISA analysis.

### Isolation and culture of human osteoarthritis articular chondrocytes and fibroblast-like synoviocytes

Human articular cartilage and synovial tissue specimens were obtained from five individuals with end-stage knee OA undergoing total knee replacement. Written informed consent was obtained from all patients, and approval was provided by the Ethics Committee of The University of Tokyo. We have complied with all relevant ethical regulations. Tissues were minced and incubated with 0.3% collagenase D in DMEM supplemented with 1% PS for 1 to 6 hours at 37 °C as previously described^16^. Human articular chondrocytes or FLS were cultured in media composed of DMEM supplemented with 10% FBS and 1% PS at 37 °C with humidified 5% CO_2_. The cells between 3 and 5 passages were used for all experiments. In the assays of LPS-induced over expression of TNF and Mmp13 using human FLS, cells were simultaneously treated with 10 ng/ml LPS and various concentrations of KAG-308 for 6 hours after 8 hours-starvation to examine the minimum concentration of KAG-308 required to induce anti-inflammatory effects.

### Collection of synovial conditioned media

Synovial tissues were harvested from the intra-articular synovium of mice with OA treated with or without 3 mg/kg KAG-308, 2 weeks after surgery. The synovial tissues were obtained from six individual mice in each treatment group. To generate conditioned media (CM), synovial tissues were incubated in 1 mL of DMEM with 1% PS for 3 hours at 37 °C with humidified 5% CO_2_ as previously described^61^. The media was then removed and tissues were incubated in 400 µL of media for 3 hours. The specimens were immediately centrifuged at 500 × g for 5 minutes, and the supernatant was aliquoted as CM and stored at −80 °C until assayed. To stimulate mouse primary chondrocytes with the CM, cells were starved for 8 hours, and co-cultured with synovial CM (150 µL) in depletion medium (250 µL) for an additional 24 hours.

### Hdac4 localization assay

To induce hypertrophic differentiation, mouse primary chondrocytes were cultured on Permanox Lab-Tek® chamber slides (Nalgene Nunc International, Rochester, NY) with DMEM supplemented with 10% FBS charcoal stripped (Thermo-Fisher Scientific; Waltham, MA), 1% PS and 100 ng/mL recombinant human bone morphogenetic protein-2 (rhBMP2) (Oriental Yeast Co; Tokyo, Japan), as previously described^37^. Cells were treated with 10 nM KAG-308, which was the concentration in cartilage in the mouse OA model treated with oral administration of 3 mg/kg KAG-308 for 4 days, following fixation in 4% paraformaldehyde for 15 minutes at room temperature and the immunohistochemistry with Hdac4 staining counterstained with DAPI. Cells with predominantly intranuclear Hdac4 were counted in 6 slides per group.

### Hypertrophic differentiation of mouse primary chondrocytes

Mouse primary chondrocytes were centrifuged at 500 × g for 5 minutes and cultured as pellets (2 × 10^5^ cells/pellet) in 96 deep well polypropylene plates (Evergreen Scientific, Vernon, CA) with DMEM supplemented with 10% FBS charcoal stripped, 1% PS and 50 µg/mL 2-Phospho-L-ascorbic acid trisodium (Sigma-Aldrich) in the presence or absence of KAG-308, as previously described^62^. The medium was replaced every other day with KAG-308. Cultured pellets were analyzed 2 weeks after treatment.

### Chondrogenesis assay with human mesenchymal stem cells from bone marrow

Human bone marrow MSC were obtained from five donors (41–65 years old) undergoing treatment with intra-articular injections of autologous MSC at Avenue Cell Clinic in accordance with a protocol approved by the Institutional Ethics Committee. Human MSC around passage 4–5 were induced to undergo chondrogenic differentiation. For the pellet culture, 2 × 10^5^ cells were placed in 96 deep well polypropylene plates and centrifuged at 500 × g for 5 minutes with basal media; DMEM/HG supplemented with 1% ITS premix (Corning; Corning, NY), 50 µg/mL 2-Phospho-L-ascorbic acid trisodium, 40 µg/mL L-proline (Sigma-Aldrich), 1% PS. The next day, the media was changed to chondrogenic-induction media; basal media supplemented with 10 nM dexamethasone (Wako), 10 ng/mL recombinant human transforming growth factor-β1 (rhTGF-β) (Oriental Yeast Co) and 100 ng/mL recombinant human bone morphogenetic protein-2 (rhBMP-2) in the presence or absence of KAG-308. The media and KAG-308 were changed twice a week. After 21 days, the pellets were analyzed.

### Quantitative reverse transcription polymerase chain reaction (qRT-PCR)

Total RNA was extracted using an RNeasy Mini Kit (Qiagen; Hilden, Germany). cDNA was transcribed from RNA using ReverTra Ace qPCR RT Master Mix with gDNA Remover (Toyobo; Osaka, Japan). Each PCR contained 1× THUNDERBIRD SYBR qPCR Mix (Toyobo), 0.3 mM specific primers, and 20 ng cDNA. The relative expression of each target gene was calculated using the 2^−ΔΔCt^ method^63^. Quantification analysis of mRNA was normalized with Gapdh, which was used as the housekeeping gene. All reactions were run in triplicate. The primer sequences are shown in Supplementary Table S1.

### Enzyme-linked immunosorbent assay (ELISA) of TNF

The concentration of TNF in the culture supernatants (CM) from FLS or OA synovial tissues was determined using Mouse TNF ELISA Kits (Research and Diagnostic Systems Inc; Minneapolis, MN) according to the manufacturer’s protocol. The minimum detectable dose of mouse TNF ranged from 0.36 to 7.21 pg/mL.

### Protein extraction and western blotting

Cells were lysed in buffer (M-PER or NE-PER; Thermo-Fisher Scientific) supplemented with COMPLETE protease inhibitor mixture (Roche). Total protein was quantified using the BCA Protein Assay Kit (Pierce, Rockford, IL). Equal amounts of protein were subjected to SDS-PAGE using 7.5% to 15% Tris-Glycine gradient gels, and blotted onto PVDF membranes (Bio-Rad Laboratories, Inc.; Hercules, CA). After blocking with 5% skimmed milk, membranes were incubated with primary antibodies against p-Creb1 (Ser133) (1:500, sc-91486), Creb1 (1:500, sc-377154), c-Fos (1:100, PC05, Calbiochem; La Jolla, CA), Hdac4 (1:200, sc-46672), Pcna (1:1000, #2586; Cell Signaling Technology, Danvers, MA), or Actin (1:1000, AC-74, Sigma-Aldrich) in Can Get Signal solution (Toyobo). The membranes were then incubated with HRP-conjugated secondary antibody (Promega; Fitchburg, WI), and immunoreactive bands were visualized with ECL plus (GE Healthcare; Buckinghamshire, England, UK) according to the manufacturer’s instructions. The blots were stripped by incubating for 30 minutes in stripping buffer (2% SDS, 100 mM 2-mercaptoethanol, and 62.5 mM Tris-HCl, pH 6.7) at 50 °C and reblotted with other antibodies. Original images of the immunoblots were shown in Supplementary Fig. S5. For quantification of bands, densitometry analysis was performed using ImageJ software (National Institutes of Health, Bethesda, MD, USA) as previously described^64^.

### Luciferase assays

The human chondrosarcoma cell line SW1353 (American Type Culture Collection; Manassas, VA) were seeded onto 24-well plates and co-transfected with 500 ng/well the CRE reporter vector (pGL4.32[luc2P/CRE/Hygro]) and 2 ng/well pRL-TK (Promega) as an internal control using FuGENE 6 transfection reagent (Roche). After 24 hours, cells were treated with various concentrations of KAG-308 for an additional 24 hours. Luciferase assays were performed using the PicaGene Dual SeaPansy Luminescence Kit (Toyo Ink, Tokyo, Japan) and a GloMax 96 Microplate Luminometer (Promega). Data are presented as the ratio of firefly to Renilla activities. Forskolin (Wako) was used as a positive control for *CRE* reporter gene assays.

### Proliferation assay

Cell proliferation and viability were examined using a CCK-8 assay kit (Dojindo; Tokyo, Japan) as previously described^16^. Briefly, human MSC were seeded onto a 96-well plate at a density of 5,000 cells/well, and cultured in DMEM containing 10% FBS and 1% PS. After 24 hours, KAG-308 was added at each concentration. The CCK-8 assay was then performed at 24, 48, 72, or 96 hours after the treatment with KAG-308. Cell viability was measured based on the amount of formazan dye using a microplate reader at 450 nm (Corona Electric Co.; Ibaraki, Japan).

### Statistical analyses

Data were expressed as the means ± standard deviation (SD) using JMP 13 (SAS Institute, Cary, NC). Statistical significance was evaluated using ANOVA and Welch’s t-test for comparison. For multiple comparisons, statistical significance for differences between groups was determined with a one-way ANOVA followed by Tukey’s or Dunnet’s *post hoc* test. *P* values less than 0.05 were considered significant.

## Supplementary information

Supplementary information.

## Data Availability

All data generated or analyzed during this study are included in this published article.

## References

[1] Hunter DJ, Bierma-Zeinstra S (2019). Osteoarthritis. Lancet.

[2] Schulze-Tanzil G (2019). Intraarticular Ligament Degeneration Is Interrelated with Cartilage and Bone Destruction in Osteoarthritis. Cells.

[3] Nelson AE, Allen KD, Golightly YM, Goode AP, Jordan JM (2014). A systematic review of recommendations and guidelines for the management of osteoarthritis: The chronic osteoarthritis management initiative of the U.S. bone and joint initiative. Semin. Arthritis Rheum..

[4] McAlindon TE (2014). OARSI guidelines for the non-surgical management of knee osteoarthritis. Osteoarthr. Cartil..

[5] Glasson SS (2005). Deletion of active ADAMTS5 prevents cartilage degradation in a murine model of osteoarthritis. Nature.

[6] Kamekura S (2005). Osteoarthritis development in novel experimental mouse models induced by knee joint instability. Osteoarthr. Cartil..

[7] Blaney Davidson EN, van Caam AP, van der Kraan PM (2017). Osteoarthritis year in review 2016: biology. Osteoarthr. Cartil..

[8] Kamekura S (2006). Contribution of runt-related transcription factor 2 to the pathogenesis of osteoarthritis in mice after induction of knee joint instability. Arthritis Rheum..

[9] Saito T (2010). Transcriptional regulation of endochondral ossification by HIF-2alpha during skeletal growth and osteoarthritis development. Nat. Med..

[10] Kobayashi H (2013). Transcriptional induction of ADAMTS5 protein by nuclear factor-κB (NF-κB) family member RelA/p65 in chondrocytes during osteoarthritis development. J. Biol. Chem..

[11] Saklatvala J (1986). Tumour necrosis factor alpha stimulates resorption and inhibits synthesis of proteoglycan in cartilage. Nature.

[12] Cheleschi S (2015). Chondroprotective effect of three different classes of anti-inflammatory agents on human osteoarthritic chondrocytes exposed to IL-1β. Int. Immunopharmacol..

[13] Chen J (2017). Efficacy and Safety of Tanezumab on Osteoarthritis Knee and Hip Pains: A Meta-Analysis of Randomized Controlled Trials. Pain. Med..

[14] Yahara Y (2016). Pterosin B prevents chondrocyte hypertrophy and osteoarthritis in mice by inhibiting Sik3. Nat. Commun..

[15] Zweers MC (2011). Celecoxib: considerations regarding its potential disease-modifying properties in osteoarthritis. Arthritis Res. Ther..

[16] Murahashi Y (2018). Intra-articular administration of IκBα kinase inhibitor suppresses mouse knee osteoarthritis via downregulation of the NF-κB/HIF-2α axis. Sci. Rep..

[17] Jia T, Qiao J, Guan D, Chen T (2017). Anti-Inflammatory Effects of Licochalcone A on IL-1β-Stimulated Human Osteoarthritis Chondrocytes. Inflammation.

[18] Cheleschi S (2018). *In vitro* comprehensive analysis of VA692 a new chemical entity for the treatment of osteoarthritis. Int. Immunopharmacol..

[19] Attur M (2008). Prostaglandin E2 exerts catabolic effects in osteoarthritis cartilage: evidence for signaling via the EP4 receptor. J. Immunol..

[20] Li X (2009). PGE2 and its cognate EP receptors control human adult articular cartilage homeostasis and are linked to the pathophysiology of osteoarthritis. Arthritis Rheum..

[21] Jónasdóttir HS (2017). Targeted lipidomics reveals activation of resolution pathways in knee osteoarthritis in humans. Osteoarthr. Cartil..

[22] Hardy MM (2002). Cyclooxygenase 2-dependent prostaglandin E2 modulates cartilage proteoglycan degradation in human osteoarthritis explants. Arthritis Rheum..

[23] Sahap Atik O (1990). Leukotriene B4 and prostaglandin E2-like activity in synovial fluid in osteoarthritis. Prostaglandins Leukot. Essent. Fat. Acids.

[24] Tchetina EV, Di Battista JA, Zukor DJ, Antoniou J, Poole AR (2007). Prostaglandin PGE2 at very low concentrations suppresses collagen cleavage in cultured human osteoarthritic articular cartilage: this involves a decrease in expression of proinflammatory genes, collagenases and COL10A1, a gene linked to chondrocyte hypertrophy. Arthritis Res. Ther..

[25] Narumiya S, Sugimoto Y, Ushikubi F (1999). Prostanoid receptors: structures, properties, and functions. Physiol. Rev..

[26] Mastbergen SC, Bijlsma JW, Lafeber FP (2008). Synthesis and release of human cartilage matrix proteoglycans are differently regulated by nitric oxide and prostaglandin-E2. Ann. Rheum. Dis..

[27] Woodward DF, Jones RL, Narumiya S (2011). International Union of Basic and Clinical Pharmacology. LXXXIII: classification of prostanoid receptors, updating 15 years of progress. Pharmacol. Rev..

[28] Regan JW (1994). Cloning of a novel human prostaglandin receptor with characteristics of the pharmacologically defined EP2 subtype. Mol. Pharmacol..

[29] Yokoyama U, Iwatsubo K, Umemura M, Fujita T, Ishikawa Y (2013). The prostanoid EP4 receptor and its signaling pathway. Pharmacol. Rev..

[30] Ho ATV (2017). Prostaglandin E2 is essential for efficacious skeletal muscle stem-cell function, augmenting regeneration and strength. Proc. Natl Acad. Sci. USA.

[31] Miyamoto M (2003). Simultaneous stimulation of EP2 and EP4 is essential to the effect of prostaglandin E2 in chondrocyte differentiation. Osteoarthr. Cartil..

[32] Weinreb M, Grosskopf A, Shir N (1999). The anabolic effect of PGE2 in rat bone marrow cultures is mediated via the EP4 receptor subtype. Am. J. Physiol..

[33] Nishitani K (2010). PGE2 Inhibits MMP Expression by Suppressing MKK4–JNK MAP Kinase–c-JUN Pathway via EP4 in Human Articular Chondrocytes. J. Cell Biochem..

[34] Fushimi K, Nakashima S, You F, Takigawa M, Shimizu K (2007). Prostaglandin E2 downregulates TNF-alpha-induced production of matrix metalloproteinase-1 in HCS-2/8 chondrocytes by inhibiting Raf-1/MEK/ERK cascade through EP4 prostanoid receptor activation. J. Cell Biochem..

[35] Nakase H (2010). Effect of EP4 agonist (ONO-4819CD) for patients with mild to moderate ulcerative colitis refractory to 5-aminosalicylates: a randomized phase II, placebo-controlled trial. Inflamm. Bowel Dis..

[36] Watanabe Y (2015). KAG-308, a newly-identified EP4-selective agonist shows efficacy for treating ulcerative colitis and can bring about lower risk of colorectal carcinogenesis by oral administration. Eur. J. Pharmacol..

[37] Kozhemyakina E, Cohen T, Yao TP, Lassar AB (2009). Parathyroid hormone-related peptide represses chondrocyte hypertrophy through a protein phosphatase 2A/histone deacetylase 4/MEF2 pathway. Mol. Cell Biol..

[38] Kozhemyakina E, Lassar AB, Zelzer E (2015). A pathway to bone: signaling molecules and transcription factors involved in chondrocyte development and maturation. Development.

[39] Chen C (2016). Compression regulates gene expression of chondrocytes through HDAC4 nuclear relocation via PP2A-dependent HDAC4 dephosphorylation. Biochim. Biophys. Acta.

[40] Sasagawa S (2012). SIK3 is essential for chondrocyte hypertrophy during skeletal development in mice. Development.

[41] Koga K (2009). Cyclic adenosine monophosphate suppresses the transcription of proinflammatory cytokines via the phosphorylated c-Fos protein. Immunity.

[42] Barry F, Murphy M (2013). Mesenchymal stem cells in joint disease and repair. Nat. Rev. Rheumatol..

[43] Caplan AI (2007). Adult mesenchymal stem cells for tissue engineering versus regenerative medicine. J. Cell Physiol..

[44] Murphy JM (2002). Reduced chondrogenic and adipogenic activity of mesenchymal stem cells from patients with advanced osteoarthritis. Arthritis Rheum..

[45] Kojima F (2009). Prostaglandin E2 activates Rap1 via EP2/EP4 receptors and cAMP-signaling in rheumatoid synovial fibroblasts: involvement of Epac1 and PKA. Prostaglandins Other Lipid Mediat..

[46] Wang P (2013). Fluid shear stress-induced osteoarthritis: roles of cyclooxygenase-2 and its metabolic products in inducing the expression of proinflammatory cytokines and matrix metalloproteinases. FASEB J..

[47] Takayama K (2002). Prostaglandin E2 suppresses chemokine production in human macrophages through the EP4 receptor. J. Biol. Chem..

[48] Zhong WW, Burke PA, Drotar ME, Chavali SR, Forse RA (1995). Effects of prostaglandin E2, cholera toxin and 8-bromo-cyclic AMP on lipopolysaccharide-induced gene expression of cytokines in human macrophages. Immunology.

[49] Mathiessen A, Conaghan PG (2017). Synovitis in osteoarthritis: current understanding with therapeutic implications. Arthritis Res. Ther..

[50] Ayral X, Pickering EH, Woodworth TG, Mackillop N, Dougados M (2005). Synovitis: a potential predictive factor of structural progression of medial tibiofemoral knee osteoarthritis–results of a 1 year longitudinal arthroscopic study in 422 patients. Osteoarthr. Cartil..

[51] Kobayashi H (2016). Biphasic regulation of chondrocytes by Rela through induction of anti-apoptotic and catabolic target genes. Nat. Commun..

[52] Chang SH (2019). Excessive mechanical loading promotes osteoarthritis through the gremlin-1-NF-κB pathway. Nat. Commun..

[53] Arnold MA (2007). MEF2C transcription factor controls chondrocyte hypertrophy and bone development. Dev. Cell.

[54] Gosset M, Berenbaum F, Thirion S, Jacques C (2008). Primary culture and phenotyping of murine chondrocytes. Nat. Protoc..

[55] Krenn V (2006). Synovitis score: discrimination between chronic low-grade and high-grade synovitis. Histopathology.

[56] Glasson SS, Chambers MG, Van Den Berg WB, Little CB (2010). The OARSI histopathology initiative - recommendations for histological assessments of osteoarthritis in the mouse. Osteoarthr. Cartil..

[57] Gao QF, Zhang XH, Yuan FL, Zhao MD, Li X (2016). Recombinant human endostatin inhibits TNF-alpha-induced receptor activator of NF-κB ligand expression in fibroblast-like synoviocytes in mice with adjuvant arthritis. Cell Biol. Int..

[58] McGarry T, Biniecka M, Veale DJ, Fearon U (2018). Hypoxia, oxidative stress and inflammation. Free. Radic. Biol. Med..

[59] Wang F (2014). Prostaglandin E-prostanoid4 receptor mediates angiotensin II-induced (pro)renin receptor expression in the rat renal medulla. Hypertension.

[60] Kondo M (2013). IL-17 inhibits chondrogenic differentiation of human mesenchymal stem cells. PLoS One.

[61] Eymard F (2017). Knee and hip intra-articular adipose tissues (IAATs) compared with autologous subcutaneous adipose tissue: a specific phenotype for a central player in osteoarthritis. Ann. Rheum. Dis..

[62] Kato Y, Iwamoto M, Koike T, Suzuki F, Takano Y (1988). Terminal differentiation and calcification in rabbit chondrocyte cultures grown in centrifuge tubes: regulation by transforming growth factor beta and serum factors. Proc. Natl Acad. Sci. USA.

[63] Schmittgen TD, Livak KJ (2008). Analyzing real-time PCR data by the comparative C(T) method. Nat. Protoc..

[64] Kumar S (2013). Autophagy triggered by magnolol derivative negatively regulates angiogenesis. Cell Death Dis..

