# Supplementary material for: Oral administration of EP4-selective agonist KAG-308 suppresses mouse knee osteoarthritis development through reduction of chondrocyte hypertrophy and TNF secretion
Source: Sci Rep. 2019 Dec 30;9:20329. doi: 10.1038/s41598-019-56861-6 (PMC6937271; doi:10.1038/s41598-019-56861-6)
Supplement: Supplementary file 1 — Supplementary information. [file 41598_2019_56861_MOESM1_ESM.docx]

**Supplementary Figures and Table**

**Oral administration of EP4-selective agonist KAG-308 suppresses mouse knee osteoarthritis development through reduction of chondrocyte hypertrophy and TNF secretion**

Yasutaka Murahashi^1,3^, Fumiko Yano^2^, Ryota Chijimatsu^1^, Hideki Nakamoto^1^, Yuji Maenohara^1^, Masahiro Amakawa^4^, Yoshihide Miyake^4^, Hiroyuki Yamanaka^5^, Kousuke Iba^3^, Toshihiko Yamashita^3^, Sakae Tanaka^1^, and Taku Saito^1^*

^1^Sensory & Motor System Medicine, ^2^Bone and Cartilage Regenerative Medicine, Graduate School of Medicine, The University of Tokyo, 7-3-1 Hongo, Bunkyo-ku, Tokyo 113-8655, Japan. ^3^Department of Orthopaedic Surgery, Sapporo Medical Univ. School of Medicine, S-1, W-16, Chuo-ku, Sapporo, 060-8543, Hokkaido, Japan. ^4^Research Planning & Collaboration Department, Drug Research Center, Kaken Pharmaceutical Co., Ltd, 14 Shinomiya, Minamigawara-cho, Yamashina-ku, Kyoto 607-8042, Japan. ^5^Pharmacokinetics and Safety Department, Drug Research Center (Shizuoka), Kaken Pharmaceutical Co., Ltd, 301 Gensuke, Fujieda, Shizuoka 426-8646, Japan


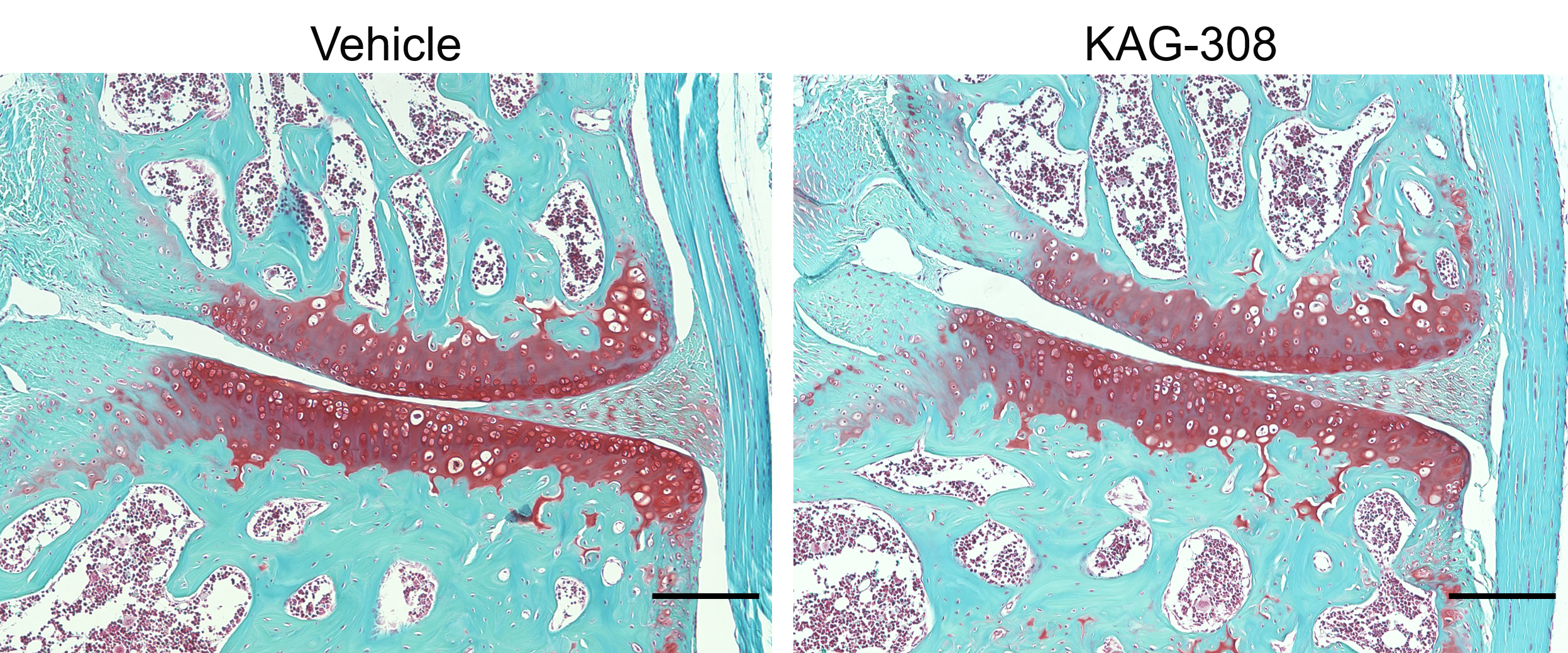


**Supplementary Figure S1.** Representative safranin-O staining of contralateral knees from the vehicle and 3 mg/kg KAG-308 groups. Scale bar, 100 µm.


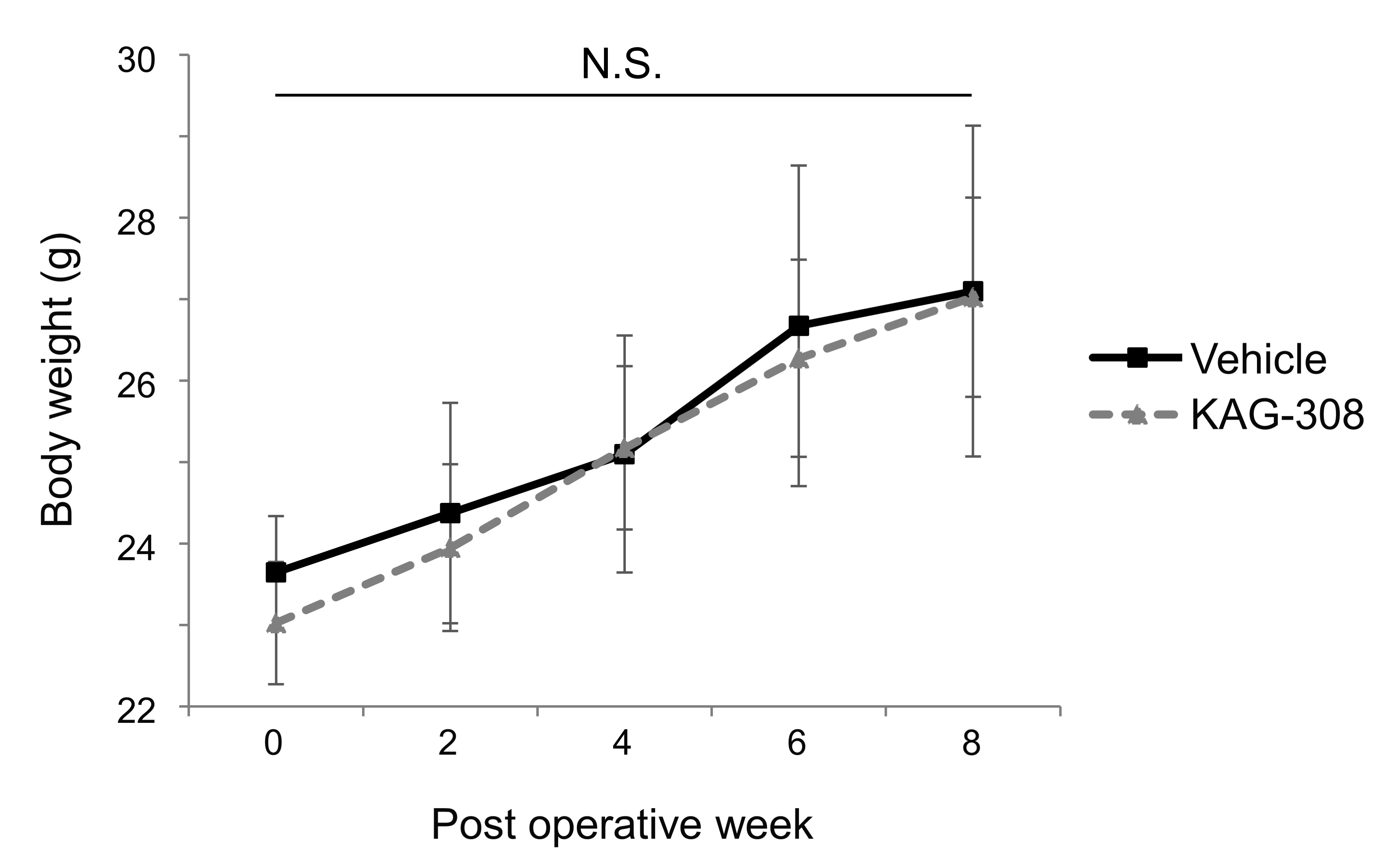


**Supplementary Figure S2.** Body weights of mice during KAG-308 treatment. Statistical significance was evaluated using Welch’s t-test for each time points. Data are expressed as the mean of 8 mice per group. Error bars indicate SD.

**Supplementary Figure S3.** Histological changes of synovium in surgically induced mouse OA knee joints. Representative HE staining of sagittal knee sections were displayed. Inset boxes in the top panels indicate the regions of the bottom panels. Scale bar, 200 µm.


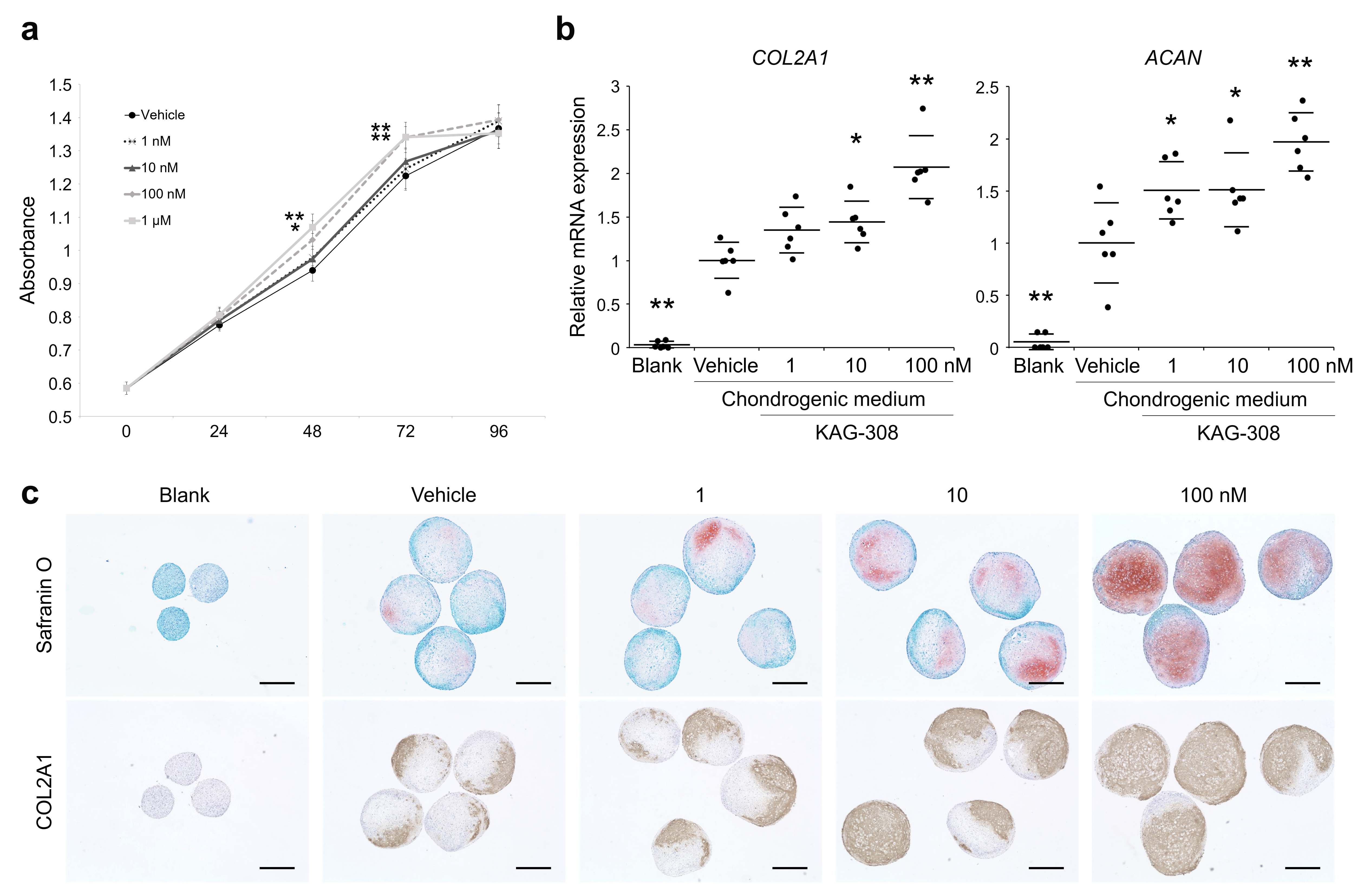


**Supplementary Figure S4.** Effects of KAG-308 on human MSC. (**a**) Proliferation of human MSC treated with KAG-308 determined by CCK-8 assay. Data are expressed as the mean of three wells per group. Error bars indicate SD. **P* < 0.05, ***P* < 0.005 vs vehicle at each time point by the ANOVA followed by Dunnett’s *post hoc* test. mRNA levels of *COL2A1* and *ACAN* (**b**), safranin-O staining, and COL2A1 immunohistochemistry (**c**) of in human MSC treated with KAG-308 after 3-wk-pellet culture in chondrogenic medium. Symbols represent individual cultured pellets; long and short bars show the mean and SD of six pellets per group, respectively. **P* < 0.05, ***P* < 0.0005 vs vehicle by ANOVA followed by Dunnett’s *post hoc* test. Scale bar, 500 µm.


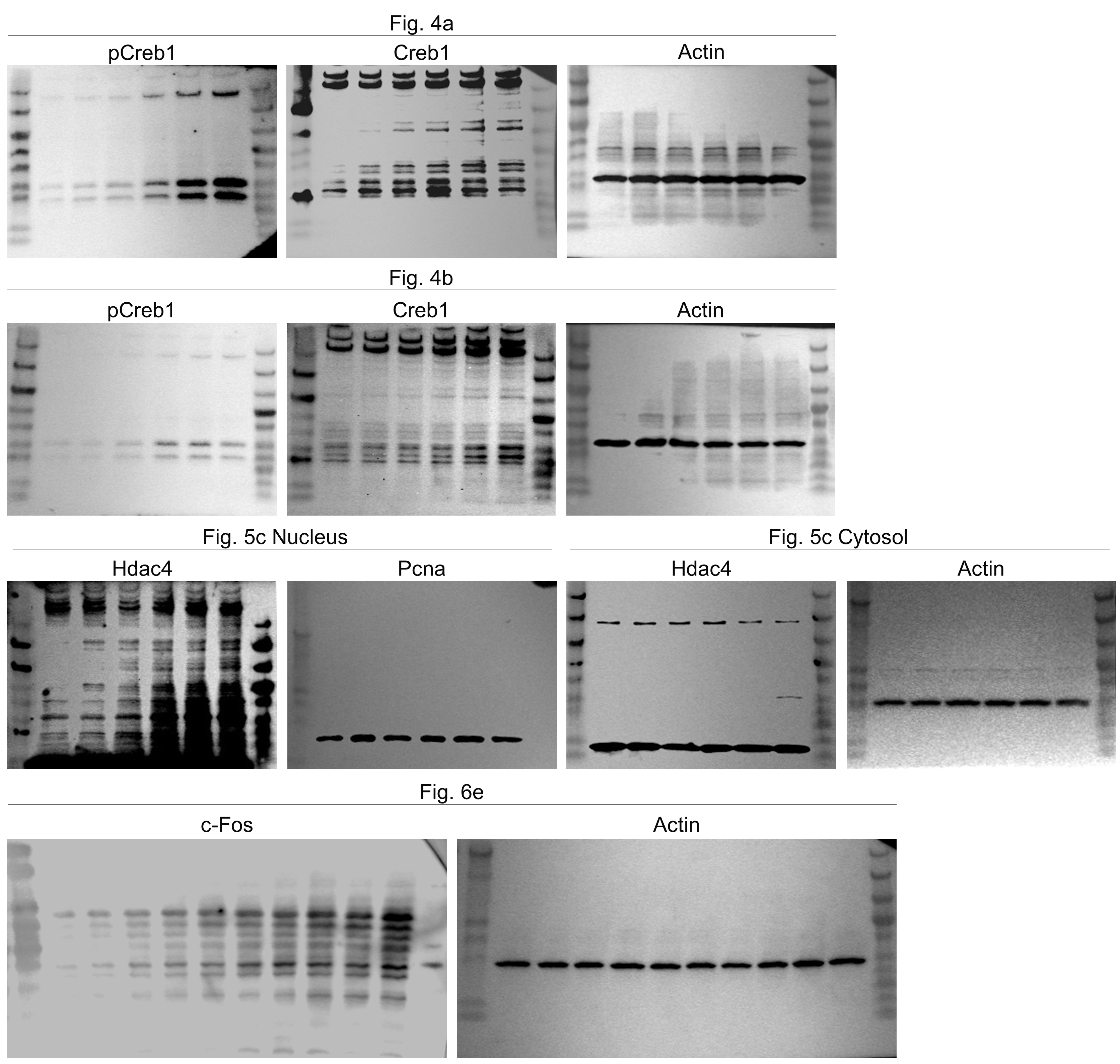


**Supplementary Figure S5.** Original images of the immunoblots shown in Fig. 4a, 4b, 5e and 6a.


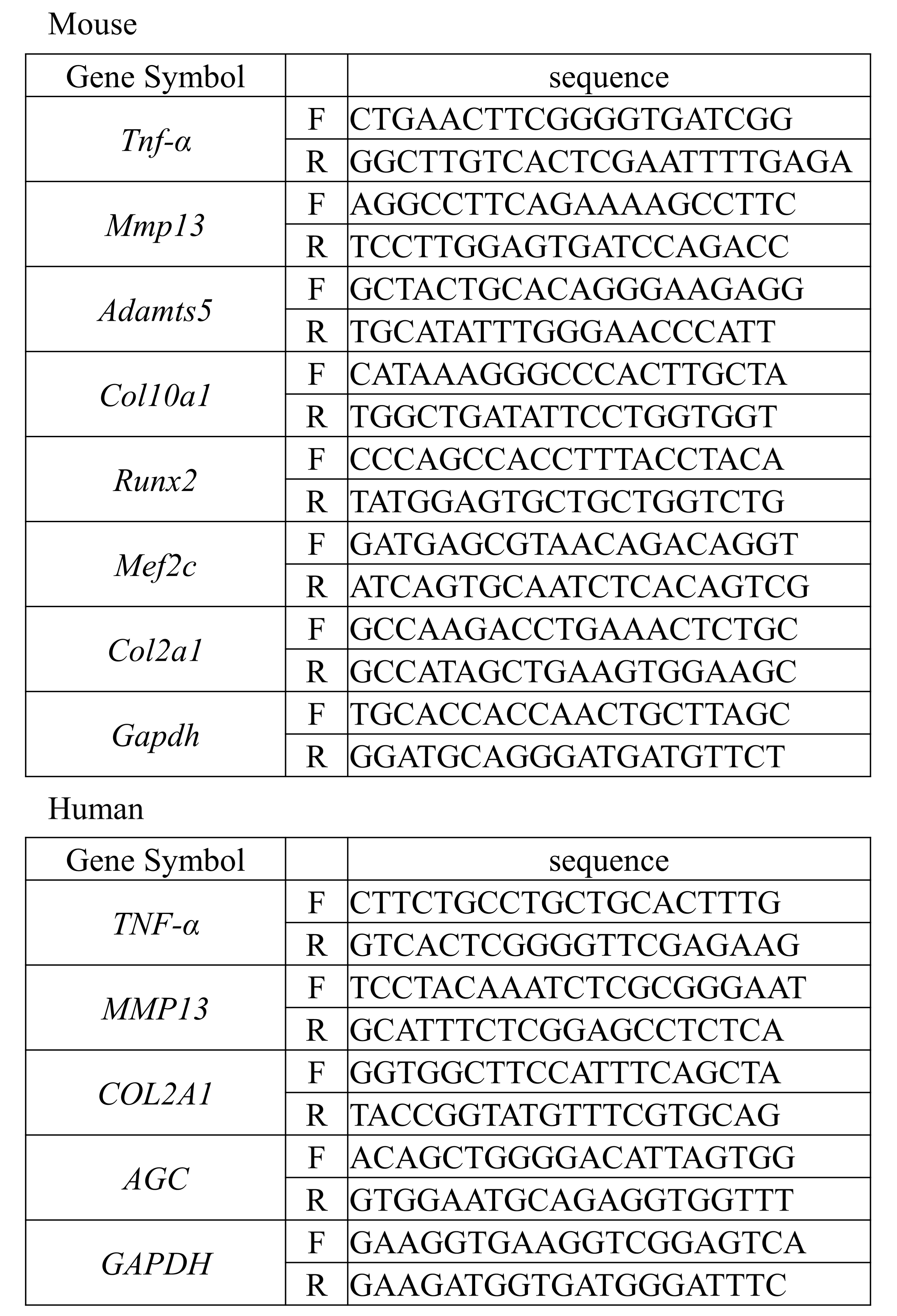


**Supplementary Table S1.** Primers used for qRT-PCR.
